# Spatial distribution and source apportionment of heavy metal(loid)s in urban topsoil in Mianyang, Southwest China

**DOI:** 10.1038/s41598-022-14695-9

**Published:** 2022-06-21

**Authors:** Huaming Du, Xinwei Lu

**Affiliations:** 1 School of Resource and Environment Engineering, Mianyang Normal University, Mianyang, 621000 China; 2 Department of Environmental Science, School of Geography and Tourism, Shaanxi Normal University, Xi’an, 710119 China

**Keywords:** Environmental sciences, Solid Earth sciences

## Abstract

Spatial distributions and sources of some commonly concerned heavy metal(loid)s (HMs, As, Ba, Cr, Co, Cu, Ni, Pb, Mn, Zn, and V) in topsoil of Mianyang city, a typical medium-sized emerging industrial city in Southwest China, were determined to explore the influences of anthropogenic activities on the urban environment. The contents of the 10 HMs in 101 topsoil samples were analyzed using an X-ray fluorescence spectrometer, and their sources were analyzed by positive matrix factorization and statistical analysis. The spatial distributions of the HMs and the source contributions were mapped using GIS technology. The results showed that the mean contents of Ba, Cr, Cu, and Zn in the topsoil were significantly higher than their background values. Industrial activities resulted in high contents of Ba, Zn, Cu, and Cr. As, Co, Ni, and V that primarily came from natural sources; Pb, Cr, Cu, and Zn were chiefly derived from a mixed source of industry and traffic; and Ba and Mn primarily originated from industrial sources. Natural sources, mixed sources, and industrial sources contributed 32.6%, 34.4%, and 33.0% of the total HM contents, respectively. Industrial sources and mixed sources of industry and traffic were the main anthropogenic sources of HMs in the urban topsoil and should be the focus of pollution control.

Urban soil plays a critical role in the healthy development of urban ecosystems. In the process of rapid urban development, heavy metal(loid)s (HMs) generated by human activities enter urban ecosystems in many ways^1^, leading to an increase in HMs in urban topsoil. HMs in topsoil can enter the human body directly or indirectly by means of ingestion, dermal contact, and inhalation^2^. Studies have shown that HMs in the human body can result in the inactivation of proteins and enzymes^3,4^ and are not easy to metabolize. When the concentration of HMs in human organs exceeds the tolerance limit, it will cause acute poisoning, subacute poisoning, and chronic poisoning, seriously endangering human health^5,6^. Thus, it is of great significance to research the concentration of HMs in urban topsoil.

Many studies have been carried out on the content, spatial distribution, current status and sources of HMs in urban topsoil. Urban topsoil often contains many more HMs than rural soil^7,8^. There are significant diversities in HM concentrations in the topsoil of different cities due to differences in soil parent material, population, traffic volume, urban scale, types of human activities and intensity of human activities^9,10^. The concentrations of HMs in urban topsoil are associated with atmospheric deposition^11^, industrial discharges^12^, automobile exhaust emissions^13^, garbage disposal^14^, abuse of fertilizers and pesticides^15^, and sewage irrigation^16^. The spatial distribution of HMs in urban topsoil varies greatly^17,18^ and poses a moderate or high risk to the ecological environment in many reported cities (e.g., Panghal et al.^19^, Rizo et al.^20^). To date, numerous studies have been implemented on heavy metal pollution in urban topsoil; nevertheless, the existing studies have been mainly concentrated on large cities (e.g., Li et al.^21^, Xiang et al.^22^) and medium-sized heavy industrial cities (e.g., Han et al.^23^, Wang et al.^24^), while research on medium-sized emerging industrial cities dominated by high-tech industries is limited. To reveal the status of HM pollution in medium-sized emerging cities and the impact of industrial activities on the urban environment, it is necessary to study the HM pollution in urban topsoil.

Mianyang is a typical medium-sized emerging industrial city in Southwest China and the second largest economy in Sichuan Province. With the acceleration of urbanization, the pressure on the urban soil environment in Mianyang city is increasing, and the problems of urban soil environmental quality and sustainable development have attracted much concern^25,26^. To the best of our knowledge, research on heavy metal pollution in Mianyang topsoil is lacking. Hence, this study aimed to determine the contents of ten HMs (viz. As, Pb, Cr, Ni, Co, Mn, V, Cu, Zn, and Ba) in the topsoil of Mianyang city and investigate their spatial distribution characteristics and sources. The results of this study will provide an important reference for the improvement of soil environmental quality and the prevention and control of HMs in Mianyang city and benefit the environmental planning and soil environmental protection of medium-sized emerging industrial cities.

## Materials and methods

### Study area

Mianyang city (30° 42′–33° 03′ N and 103° 45′–105° 43′ E) is situated in northeastern Sichuan Province^27^. The terrain is high in the northwest and low in the southeast^27^, with an altitude of 275–4888 m. Mianyang has a humid monsoon climate in the north subtropical mountains, with an annual precipitation of 545.50–1699.70 mm and an annual average temperature of 15.40–18.13 °C^28^. The dominant wind directions are southeast and north^28^. The Fujiang River and Anchang River traverse the study area, and the main soil types are loess and cinnamon. Mianyang has developed rapidly in the past 20 years: the built-up area, urban population, regional GDP, number of industrial enterprises, and number of vehicles in Mianyang (municipal districts) have increased from 44 to 163 km^2^, 1.04–1.75 million, 139 billion yuan to 1592 billion yuan, 138–547, and 0.12–1.01 million, respectively^29^. The east of the study area is industrial, the area near the river junction is residential and mixed commercial use, the northwest is a technological industrial area with electronics, machinery, and photoelectric technology, the center is a comprehensive science and educational area, and the west is a high-tech industrial zone integrating machinery, plastics, energy, electronics, pharmaceuticals, and photoelectric technology.

### Soil sampling and experimental analysis

**Figure 1:**
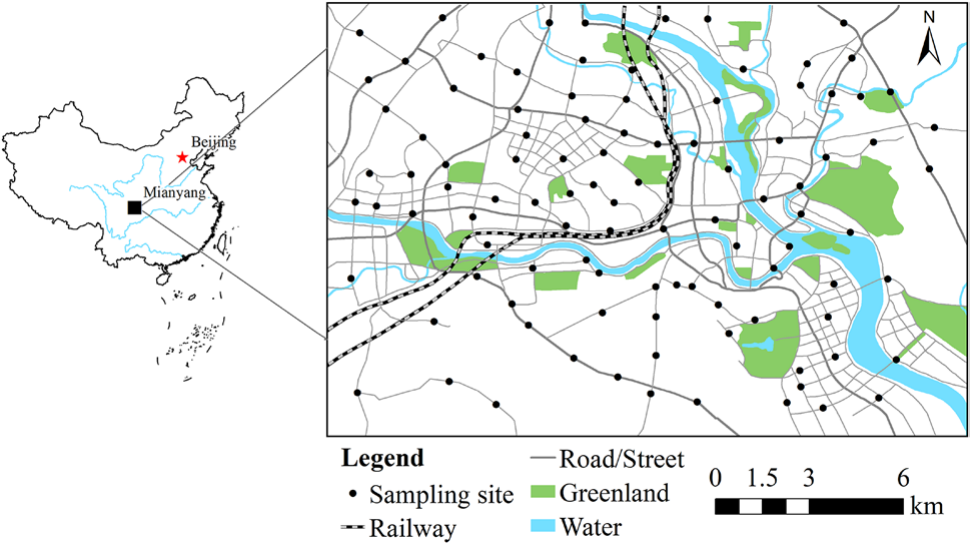
Research region and sampling locations in Miangyang, China [the figure was generated by Huaming Du using the ArcGIS 10.3 (https://developers.arcgis.com/)].

Following the principles of objectivity, accuracy, balance, and representativeness of the sampling site layout^30^, combined with the size of the research area, the study area was divided into 1700 × 1700 m grids by the grid method. In the process of sampling, the positions of the actual sampling sites were appropriately adjusted according to the actual situation of the investigation area. Figure 1 shows the locations of the sampling sites. In August 2021, 101 topsoil samples (0–20 cm depth) were collected from the road green belts of Mianyang with a Luoyang shovel. During the sampling process, the samples were accurately located by using a global positioning system, and the actual characteristics of the sampling site were recorded. At each sampling site, five topsoil samples were collected from the green belt along the main road and mixed into a composite sample of approximately 1.5 kg^31^. After removing rocks, grass roots and garbage^31^, the collected topsoil samples were placed in plastic bags, marked with numbers and sampling information, and then brought back to the laboratory for analysis.

The topsoil samples were placed on white porcelain plates and air-dried in a dark, ventilated laboratory. A 1.0-mm nylon sieve was used to sieve the air-dried soil samples, and then some sieved topsoil samples were taken for physical–chemical property determination. Approximately 30 g of residual topsoil was extracted from every screened topsoil sample and then ground with a vibration grinder until all samples passed through a 0.075-mm nylon sieve^32^. The milled topsoil sample (5.0 g) and plastic ring (inner diameter 34 mm) were put into a mold, and then the tablet was compressed by a tablet press under 30 t pressure. Finally, the contents of arsenic (As), chromium (Cr), cobalt (Co), copper (Cu), lead (Pb), manganese (Mn), nickel (Ni), zinc (Zn), barium (Ba), and vanadium (V) in the topsoil of Mianyang were examined by using an X-ray fluorescence spectrometer (XRF, Bruker, S8 Tiger, Germany), and quality control was performed using 10% repeated samples and the standard samples (GSS-2). The error of the examined HMs was < 5%.

### Ordinary kriging interpolation

Due to the spatial autocorrelation of the heavy metal content data, based on the spatial autocorrelation, using the semivariance function structure of the original data, ordinary kriging interpolation (OK) was chosen to estimate the regionalized variable value of unknown sampling points without bias. The formula of the OK method is as follows^33^:

1 $$ {\text{Z}}^{ * } (x_{0} ) = \sum\limits_{i = 1}^{n} {\lambda_{i} } Z(x_{i} ), $$

where Z^*^(*x*_0_) is the estimated value of point *x*_0_, *λ*_*i*_ is the weight of the estimated point element, *Z*(*x*_*i*_) is the detected value of the known sampling point *x*_*i*_, and *n* is the number of actual sampling points. OK was applied to analyze the spatial distribution of HMs and different pollution sources in the Mianyang urban area.

### Correlation analysis

Correlation analysis refers to the analysis of two or more variables that have correlations to measure the degree of correlation between different variables. Spearman correlation, proposed by Spearman^34^, is widely used to reflect the degree of correlation between variables because of its simplicity, robustness and natural properties^35^. Spearman correlation analysis was used to detect whether the 10 HMs were correlated.

### Principal component analysis

Principal component analysis (PCA) is a kind of multivariate statistical analysis method used to remove data redundancy. It mainly uses the concept of dimensionality reduction to eliminate the overlapping parts among many elements^36^. By using the principle of maximum variance, several independent variables contained in the original data are fitted linearly, and the new low-dimensional variables are substituted for the original high-dimensional variables to achieve the goal of data dimension reduction. PCA was applied to identify the sources of HMs.

### Cluster analysis

Cluster analysis (CA) is a statistical method that can be used to study classification. Cluster analysis is used to classify variables according to their similarity in attributes or degree of affinity, and the samples are then clustered according to the degree of closeness^37^. In this paper, 10 HMs were analyzed by using SPSS software, Euclidian distance was used to calculate the cluster distance between samples, and the average weighted method was used to calculate the system cluster tree. CA was used to determine the sources of HMs.

### Positive matrix factorization

Positive matrix factorization (PMF), proposed by Paatero and Tapper^38^, is one of the source analysis methods suggested by the U.S. EPA^39^ and is an effective source apportionment method^40^. The computational model is described as shown in Eq. (2)^39^:

2 $$ X_{ij} = \sum\limits_{k = 1}^{p} {G_{ik} } F_{kj} + E_{ij} , $$

where *X*_*ij*_ is the concentration of HM *j* in sample *i*, *G*_*ij*_ is the contribution of source *k* to sample *i*, *F*_*ij*_ is the concentration of HM *j* in source *k*, and *E*_*ij*_ is the residual matrix. The optimal matrices *G* and *F* were obtained by continuous decomposition of the original matrix *X* with the least squares. The ultimate goal was to minimize *Q*. *Q* was calculated using Eq. (3)^39^:

3 $$ Q = \sum\limits_{i = 1}^{n} {} \sum\limits_{j = 1}^{m} {\left( {\frac{{e_{ij} }}{{u_{ij} }}} \right)}^{2} , $$

where *u*_*ij*_ is the uncertainty of HM *j* in sample i. The PMF model requires an input uncertainty file. The uncertainty data file is calculated as follows.

When the content of HMs is lower and higher than the detection limit (MDL), the uncertainty is calculated by Eq. (4)^39^ and Eq. (5)^39^, respectively:

4 $$ U_{nc} = \frac{5}{6}MDL, $$

5 $$ U_{nc} = \sqrt {(\sigma \times c)^{2} + MDL^{2} } , $$

where *σ* is the relative standard deviation, *C* is the content of HM, and MDL is the method detection limit. The PMF model was used to analyze the contribution rate of each source to each heavy metal element.

## Results and discussion

### Content characteristics

The contents of HMs in the urban topsoil of Mianyang and the background values of Sichuan topsoil^41^ are listed in Table 1. The average contents of Ba, Cr, Cu, and Zn in topsoil were 1.2, 1.6, 1.2, and 1.2 times the reference value, respectively. The coefficient of variation (CV) is a statistical measure of the dispersion of data points in a data series around the mean. Based on the literature^42^, CV > 100% is regarded as very high variability, 51% < CV ≤ 100% is considered high variability, 21% < CV ≤ 50% indicates moderate variability, and CV ≤ 20% indicates low variability. Table 1 shows that the CV values of Co and Cu were 62.0% and 91.6%, respectively, exhibiting high variability; Pb, Cr, Zn, Mn, and As presented moderate variability in the topsoil of Mianyang, and Ba, Ni, and V exhibited low variability. These results indicate that anthropogenic activities have different influences on the HMs analyzed in the topsoil of Mianyang.

**Table 1:** Contents of HMs and background values in topsoil of Sichuan (mg kg^−1^).

Element	As	Ba	Co	Cr	Cu	Mn	Ni	Pb	Zn	V
Mean	11.2	586.8	20.2	124.7	37.8	661.5	34.9	28.4	102.2	96.6
Minimum	3.0	2926.0	7.4	72.0	16.7	71.6	16.7	16.6	40.0	42.8
Maximum	30.0	873.4	93.9	252.0	369.9	1356.9	49.6	60.0	320.2	155.5
Median	10.1	572.5	17.1	119.3	33.2	663.3	35.4	27.0	87.4	97.4
Standard deviation	4.2	110.9	12.5	28.9	34.6	168.6	5.7	7.1	39.0	15.8
Coefficient of variation (%)	37.6	18.9	62.0	23.2	91.6	25.5	16.2	24.9	38.2	16.4
Kurtosis	4.5	− 0.1	23.3	3.1	85.1	5.1	1.5	5.3	15.5	2.9
Skewness	1.7	0.1	4.5	1.3	8.9	0.8	− 0.8	1.9	3.4	− 0.2
Reference value^41^	10.4	474.0	17.6	79.0	31.1	657.0	32.6	30.9	86.5	96.0

Table 1 shows that the kurtosis values of the HMs determined in the topsoil samples, except for Ba, were > 0, implying that the content distributions of these HMs in the topsoil of Mianyang were steeper than the normal distribution^39^. The positive skewness of As, Pb, Cr, Co, Cu, Mn, Ba, and Zn indicated that their distribution had a long right tail, viz. many extreme values emerging at the right end of their distribution data, and the dispersion on the right side of the mean value was strong, which was confirmed by the mean values being higher than the median^43,44^. Ni and V were left-skewed, and their mean contents were lower than their median contents.

According to the existing reports on HM pollution in urban topsoil, combined with the similarity of industrial structures and the comparability of HMs in urban topsoil, Karamay^24^, Yinchuan^44^, Rawalpindi^45^, Klang^46^, Beni Mellal^47^, Ancona^48^, Changchun^49^, Guiyang^50^, Yan’an^51^, Xi’an^52^, Beijing^53^, and Nanjing^54^ were selected for comparison (Table 2). It was found that the mean content of V was much higher in Mianyang than in Xi’an and Yinchuan. Except for Klang, Ancona, and Xi’an, the mean content of Co in Mianyang was lower than that in other cities. The Cr content in the topsoil of Mianyang was higher than that of other cities except for Rawalpindi. The contents of Cu, As, Ni, Mn, and Zn were at moderate levels compared with the other cities listed in Table 2. Except for Beijing, Yinchuan, and Yan’an, the Pb concentrations were lower than those recorded in other cities. The differences in the HM contents in topsoil from diverse cities may be closely correlated with soil parent material, local natural environment, urban history and scale, intensity of human activities, pollution control technology and environmental management level.

**Table 2:** Comparative analysis of HM contents (mg kg^−1^) in the topsoil of diverse cities. NA means not available.

City	As	Ba	Co	Cr	Cu	Mn	Ni	Pb	Zn	V	References
Karamay	20.65	NA	NA	117.86	64.22	NA	42.7	32.56	123.32	NA	Wang et al.^24^
Yinchuan	NA	NA	37.2	109.1	16.8	NA	25.3	25.0	26.0	59.9	Zhang et al.^44^
Rawalpindi	NA	NA	33.4	295.3	335.7	633.7	235.7	1572.4	542.5	NA	Shehzad et al.^45^
Klang	NA	NA	1.20	15.58	29.35	NA	NA	52.73	275.75	NA	Yuswir et al.^46^
Beni Mellal	3.9	NA	NA	64.4	46.8	1097.9	20.2	95.1	228.6	NA	Odewande and Abimbola^47^
Ancona	NA	NA	18.1	45.6	63.9	NA	50.9	97.4	199.1	NA	Serrani et al.^48^
Changchun	12.5	NA	NA	66.0	29.4	880.0	NA	35.4	90.0	NA	Yang et al.^49^
Guiyang	16.8	NA	NA	NA	66.1	NA	38.9	79.5	243.0	NA	Li et al.^50^
Yan’an	NA	NA	NA	66.22	23.65	NA	37.56	20.18	71.20	NA	Hu et al.^51^
Xi’an	NA	NA	19.3	81.1	54.3	671.5	34.5	59.7	186.2	85.2	Chen et al.^52^
Beijing	NA	NA	NA	61.0	31.7	NA	24.0	23.3	92.9	NA	Wang et al.^53^
Nanjing	9.94	NA	NA	74.54	37.25	NA	31.89	32.52	109.15	NA	Wang et al.^54^
Mianyang	11.2	586.8	20.2	124.7	37.8	661.5	34.9	28.4	102.2	96.6	This work

### Spatial distribution characteristics

**Figure 2:**
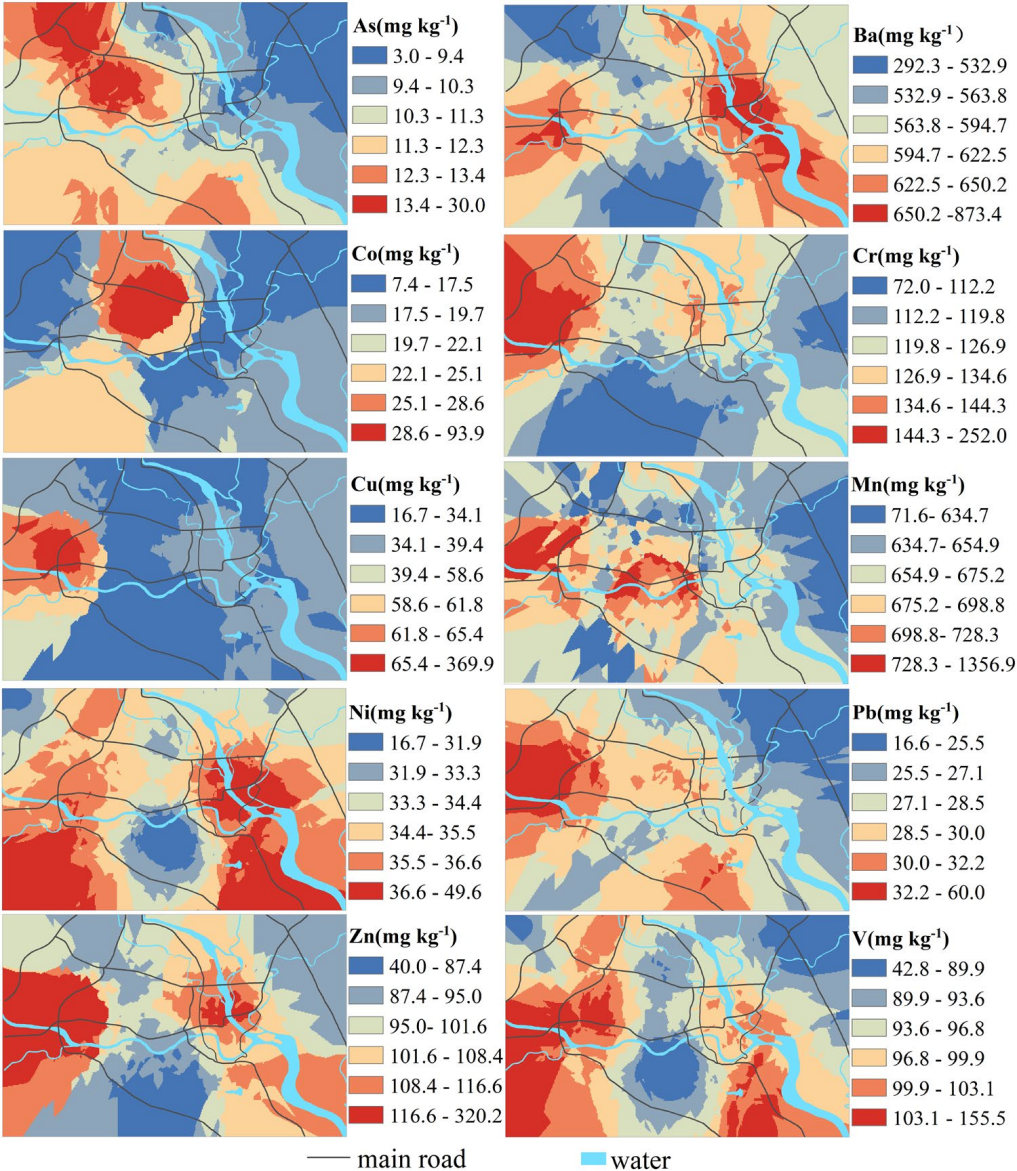
Spatial distribution of HM contents in the topsoil of Mianyang [the figure was generated by Huaming Du using the ArcGIS 10.3 (https://developers.arcgis.com/)].

Ordinary kriging interpolation in ArcGIS 10.3 was applied to detect the spatial distribution maps of the determined HMs in the topsoil of the Mianyang urban area, and the results are presented in Fig. 2. The spatial distribution of different HMs varied greatly, and the high-value areas of As with 11.3–30.0 mg kg^−1^ (1.1–2.9 times background value) mainly appeared in the western part of the study area in the science and technology industrial area and high-tech industrial zone. The spatial variations in Ba and Mn were similar. They were found in high concentrations near an industrial zone, where there were mainly stone factories, machinery factories, an automobile city, a wholesale city, and a plastic company. The concentration of Co in the topsoil of Mianyang was low; its contents were higher than its background value in only 27 samples. The high-value areas of Co with 22.1–93.9 mg kg^−1^ were mainly distributed in the middle of the north and the southwest of the study area, which were around schools and residential areas. The contents of Cr in 99 samples were higher than its background value, and its high-value areas with 126.9–252.0 mg kg^−1^ (1.6–3.2 times background value) were primarily distributed in the north and northwest of the study area. The common characteristic of the high-value areas of Cr is that there were more industries and small reprocess shops around, and these are greatly influenced by human activities. The high-value sites of Cu with 58.6–369.8 mg kg^−1^ (1.9–11.9 times the background value) were distributed in the western part of the study area, viz. the high-tech industrial zone.

The spatial variation trends of Ni and V were consistent, and their high-value sites were found in the southwestern and southeastern parts of the study area. These two HMs were distributed in strips along the river, indicating that the concentrations of Ni and V were high in the area near both banks of the river, and their mean contents were close to their background values. Pb was mainly concentrated along urban trunk roads, and it has been found that Pb particles can be released from gasoline combustion and car body wear^32^, which leads to an increase in the concentration of Pb in topsoil. In particular, a high-value area appeared in the western part of the city; this area is the only road from Mianyang to Chengdu and Deyang, and it is also the area with the largest traffic flow in the city. In general, the high-value areas of Pb with 28.5–60.0 mg kg^−1^ were mainly located in the zone with a large number of vehicles and heavy traffic in Mianyang city. The high-value area of Zn with 101.6–320.2 mg kg^−1^ (1.2–3.7 times the background value) was mainly distributed in the high-tech industrial zone in the west of the city and in industrial areas in the east of the city. There were electronics companies, electrical companies, gas companies, hospitals, plastic steel reprocessing shops, machinery companies, and energy companies in the area with high Zn values.

### Statistical analysis results

Using IBM SPSS 25.0 (IBM Corporation, Armonk, New York, USA)(https://www.ibm.com/products/spss-statistics) software, the relationship between the 10 HMs in the topsoil of Mianyang was explored by principal component analysis (PCA), cluster analysis, and Spearman correlation analysis. The Spearman correlation analysis results are displayed in Table 3. At the *P* < 0.01 level, a remarkably positive correlation existed in the following HM pairs: As–Co (0.490), As–Ni (0.467), As–Pb (0.512), As–V (0.538), Ba–Cu (0.531), Ba–Mn (0.457), Ba–Ni (0.304), Ba–Zn (0.663), Ba–V (0.303), Co–Ni (0.346), Co–Pb (0.356), Co–V (0.371), Cr–Cu (0.378), Cr–Zn (0.378), Cu–Mn (0.275), Cu–Ni (0.404), Cu–Pb (0.460), Cu–Zn (0.754), Cu–V (0.349), Ni–Zn (0.306), Ni–V (0.871), and Pb–Zn (0.323). As–Ba (− 0.261) and Co–Cr (− 0.315) showed a notably negative correlation at *P* < 0.01. The pairs Ba–Cr (0.249), Mn–Ni (0.231), Ni–Pb (0.198), Pb–V (0.241), and Zn–V (0.229) were positively correlated at *P* < 0.05, and As–Cr (− 0.235) was negatively correlated at *P* < 0.05. The significantly positive correlation between HMs indicates that they may have common sources or similar geochemical characteristics, while a significantly negative correlation indicates the opposite.

**Table 3:** Spearman correlation matrix of HMs in the urban topsoil of Mianyang. **Correlation is significant at *P* < 0.01 (2-tailed). *Correlation is significant at *P* < 0.05 (2-tailed).

	As	Ba	Co	Cr	Cu	Mn	Ni	Pb	Zn	V	Al
As	1										
Ba	− 0.261**	1									
Co	0.490**	− 0.124	1								
Cr	− 0.235*	0.249*	− 0.315**	1							
Cu	0.076	0.531**	0.08	0.378**	1						
Mn	0.111	0.457**	0.13	− 0.14	0.275**	1					
Ni	0.467**	0.304**	0.346**	− 0.154	0.404**	0.231*	1				
Pb	0.512**	− 0.023	0.356**	0.171	0.460**	0.182	0.198*	1			
Zn	− 0.172	0.663**	− 0.041	0.378**	0.754**	0.194	0.306**	0.323**	1		
V	0.538**	0.303**	0.371**	− 0.144	0.349**	0.165	0.871**	0.241*	0.229*	1	
Al	0.331**	0.126	0.267**	− 0.088	0.163	0.087	0.473**	0.105	0.07	0.501**	1
Fe	0.323**	0.081	0.296**	− 0.14	0.144	0.061	0.486**	0.03	0.063	0.485**	0.923**

The results of PCA demonstrated that there were three eigenvalues > 1, and these three principal components described 71.78% of the total variance (Table 4). Principal component 1 (PC1), which described 27.69% of the total variance, had a strong loading for As, Ni, Co, and V. Principal component 2 (PC2) was mainly loaded by Cr, Pb, Cu, and Zn, explaining 25.34% of the total variance. Principal component 3 (PC3) was mainly dominated by Ba and Mn, which moderately explained 18.75% of the total variance.

**Table 4:** Rotated component matrix for HM data in the topsoil of Mianyang.

Element	Principal Component		
	1	2	3
As	**0.783**	0.100	− 0.419
Ba	0.049	0.316	**0.868**
Co	**0.559**	− 0.144	0.027
Cr	− 0.448	**0.673**	− 0.106
Cu	0.130	**0.825**	0.180
Mn	0.022	− 0.012	**0.828**
Ni	**0.853**	0.261	0.213
Pb	0.402	**0.692**	− 0.061
Zn	0.104	**0.812**	0.394
V	**0.850**	0.251	0.107
Eigenvalue	3.454	2.302	1.423
% of variance	27.69	25.34	18.75
% of cumulative	27.69	53.03	71.78

Significant values are in bold.

**Figure 3:**
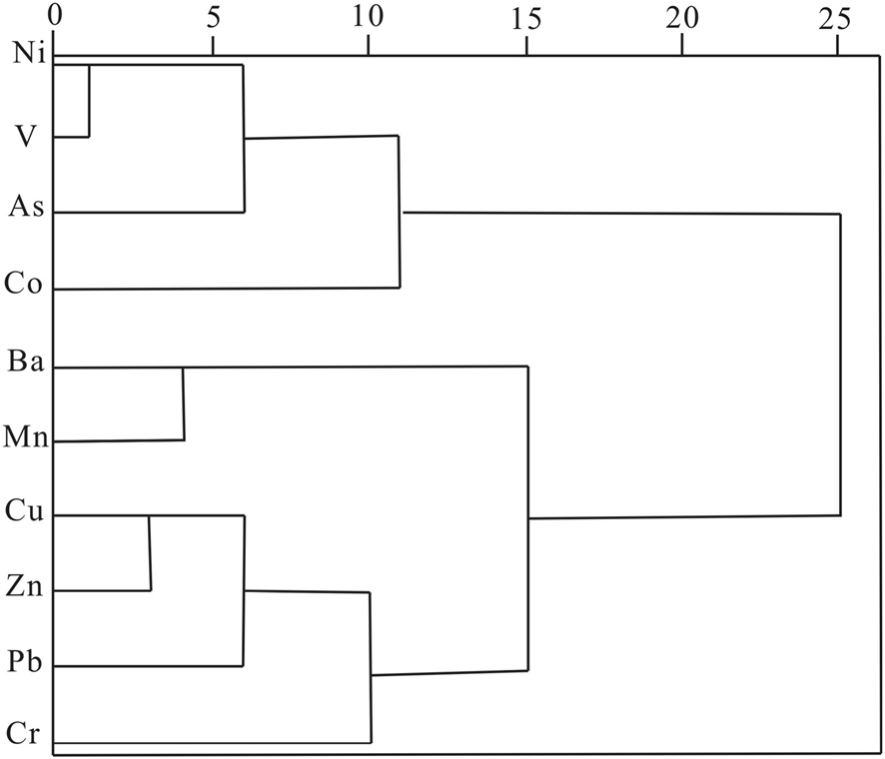
Dendrogram of cluster analysis for 10 HMs.

HM concentration data were normalized by *z*-scores before CA. The Euclidean distance method and Ward’s method were used in CA to obtain the clustering tree of 10 HMs (Fig. 3), and the clustering tree vividly reflected the relationship between the different HMs. Figure 3 shows that there were three clusters, Ni, V, As, and Co, and they belonged to cluster 1. Ba and Mn belonged to cluster 2. Cu, Zn, Pb, and Cr belonged to cluster 3. The CA results were in accordance with the PCA results.

### Source apportionment

Three main sources were identified for the HMs investigated in Mianyang topsoil according to the content and distribution of the HMs and their relationships, viz. (1) As, Co, Ni, and V principally originated from natural soil; (2) Cr, Pb, Cu, and Zn were chiefly generated from a mixed source of industry and traffic; and (3) Ba and Mn were chiefly from industrial sources.

In the aforementioned statistical analysis, As, Ni, Co, and V had remarkably positive correlations in Spearman correlation analysis (Table 3) and belonged to the same cluster in CA (Fig. 3) and the same principal component in PCA (Table 4), indicating that these four HMs in the topsoil of Mianyang may have similar sources or geochemical behaviors. The contents of As, Co, Ni, and V in 55.4–77.2% of the samples were less than or close to their background values in the Sichuan topsoil. Co and As exhibited high and moderate variability, respectively, while Ni and V exhibited low variability, indicating that the Ni, V, Co, and As in the topsoil of Mianyang were mainly controlled by natural factors, and Co and As were also affected by human activities to a certain extent. Al and Fe are conservative elements in the natural environment^42^. The correlation analysis results (Table 3) showed that Al and Fe were significantly positively correlated with these four HMs. Based on these results and related literature^55^, we deduced that the As, Ni, Co, and V in the topsoil of Mianyang probably originated from natural sources such as rock weathering and soil erosion processes.

The second group HMs, including Cu, Pb, Zn, and Cr, presented a remarkably positive correlation in Spearman correlation analysis. These four HMs were classified into a cluster (cluster 3) in CA and the same principal component (PC 2) in PCA. The statistical analysis results implied that Cu, Zn, Pb, and Cr in the topsoil of Mianyang probably had the same source or analogous geochemical behaviors^32^. The contents of Cu, Cr, and Zn in most samples (> 65%) and Pb in 22% of samples were higher than their corresponding background values. The CV values indicated that Pb, Cr, and Zn were moderately variable, while Cu was highly variable. Table 3 shows that Cu, Zn, Pb, and Cr were not related to the conservative elements Al and Fe. The aforementioned content characteristics and statistical analysis results showed that the Cu, Pb, Zn, and Cr in the topsoil of Mianyang were mainly affected by human activities. Previous studies reported that Cr is widely used in the production of stainless steel, titanium alloy, and aluminum alloy and is also used in electroplating, tanning, metal pickling industries, and automobile parts^56^. Cu in the surface environment comes from metal casting, smelting slag, spills of waste residue and other industrial activities^57^. In addition, Cu is a part of the alloys used in the production of mechanical parts^58^. Pb is often used in car lubricants and brass automotive radiators^58^. Zn is widely used in galvanizing, copper alloys, and zinc alloys, and it is also used in tires for automobiles. According to the spatial distribution of Cr in the topsoil of Mianyang, the high-value areas of Cr were mainly located near processing enterprises such as machinery plants, mechanical and electrical plants, and plastic steel doors and windows, and the content of Cr at 99 sampling sites was higher than its background value in Sichuan topsoil. The high-value sites of Cu were mainly from electronics companies, electrical appliances companies and machinery manufacturing companies. Therefore, the high concentration of Cu in topsoil may be due to machinery manufacturing and metal processing. From the spatial distribution of Zn, it can be concluded that the main source of Zn was industrial. The high content of Pb in the topsoil of Mianyang was mainly concentrated in areas with dense road networks and heavy traffic. In general, Cr, Pb, Cu, and Zn were primarily distributed in industrial areas and intensive traffic areas, which indicates that these four HMs were greatly influenced by human activities and mainly came from a mixed source of industry and traffic. This finding was consistent with previous studies, i.e., car body wear and gasoline combustion increasing the contents of Pb, Cu, and Zn in soils^32^. It is concluded that the accumulation of Cr, Cu, Pb, and Zn in the topsoil of Mianyang may be caused by the pollution of industrial production and transportation, which were mainly considered to be derived from a mixed source of industry and traffic.

The third group of HMs contained Ba and Mn. Ba was significantly positively correlated with Mn in the Spearman correlation analysis. These two HMs were classified together (cluster 2) in CA and belonged to the same principal component (PC3) in PCA. The statistical analysis results implied that these two HMs had the same origin. The level of Ba in 86% of samples and the level of Mn in 51% of samples were higher than their background values. Mn had moderate variability, and Ba had low variability in the investigated soil samples. There was no significant correlation between Ba and Mn and the conservative elements Al and Fe. The abovementioned statistical analysis results and content characteristics showed that the Ba and Mn in the topsoil of Mianyang were chiefly affected by human activities. Ba is a constituent of alloys, paint, ceramic, glass or plastic cements. Ba is also used as a cleaning agent, an oxidizing corrosive agent, and an additive to suppress black smoke in internal combustion engines^59^. Anthropogenic sources of Mn in the environment include the chemical industry, mining and smelting^60^. In view of the content, distribution, statistical analysis results, and previous studies, we conclude that the Ba and Mn in the topsoil of Mianyang mainly originated from industrial sources.

**Figure 4:**
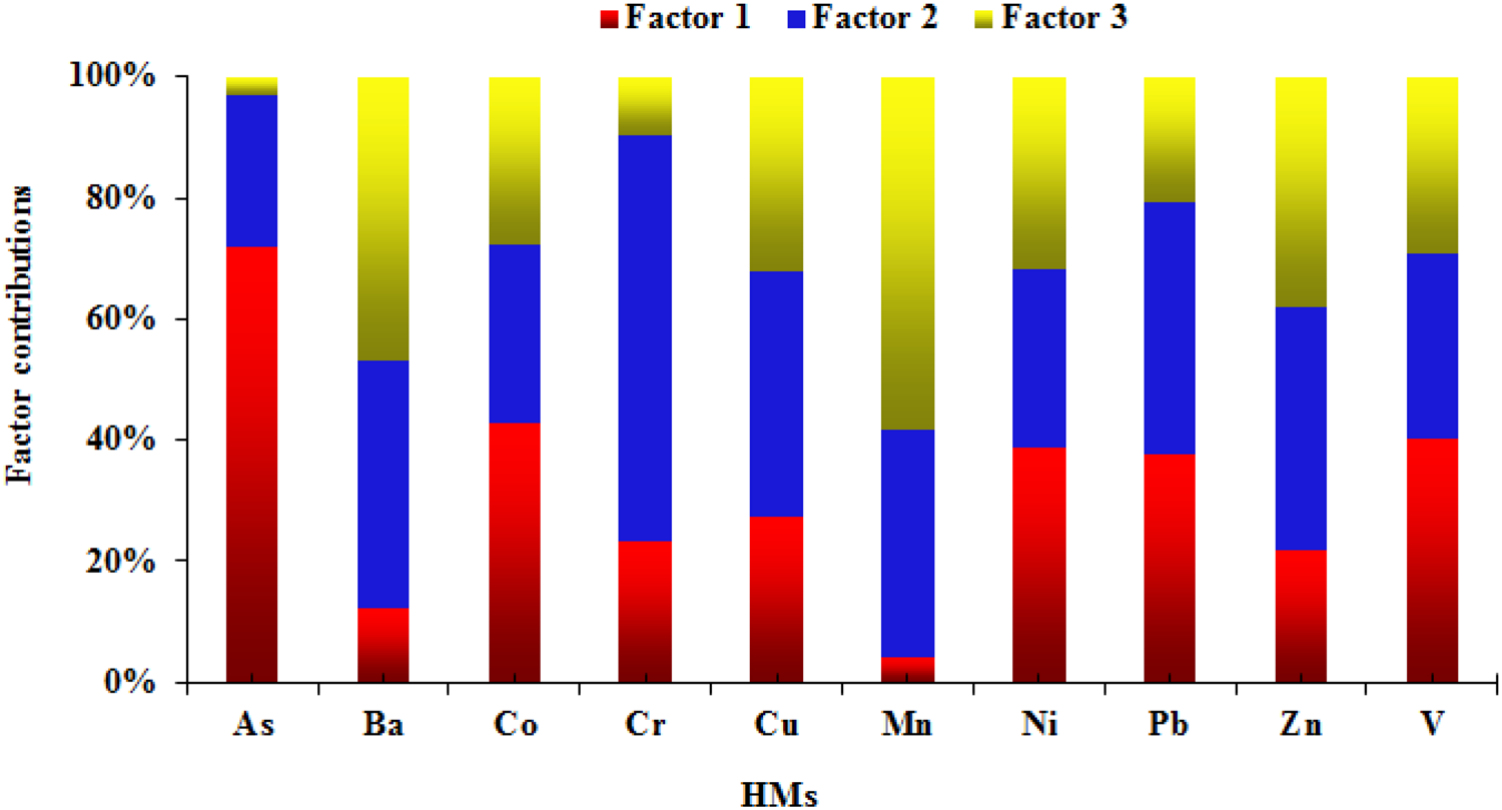
Source contribution rates of the three sources to HMs in the topsoil of Mianyang.

To ascertain the quantitative contribution of the above three identified sources to HMs in the topsoil, the EPA PMF 5.0 model was used in this study. Considering the factors of the signal-to-noise ratio (s/n), Q value, normalized residuals, and correlation coefficient *r*^2^, it was determined that the number of factors was 3 and the number of runs was 20. The source contributions of the HMs measured in the topsoil of Mianyang are shown in Fig. 4. The As, Co, V, and Ni in the topsoil of Mianyang were chiefly contributed by natural sources (Factor 1), which accounted for 71.9%, 42.9%, 40.1%, and 38.6%, respectively. Meanwhile, natural sources had a high contribution to Pb (37.5%), and the contributions to Cr, Cu, and Zn were close, viz. respectively contributing 23.4%, 27.4%, and 21.9%. The Cr, Cu, Pb, and Zn in the topsoil of Mianyang were primarily controlled by a mixed source of industry and traffic (Factor 2), contributing 66.9%, 40.4%, 42.0%, and 40.0% of these HM contents, respectively. The mixed source of industry and traffic contributed 41.0% of the Ba content. Furthermore, 25.0% of the As content, 29.3% of the Co content, 37.8% of the Mn content, 29.7% of the Ni content, and 30.6% of the V content in the topsoil were contributed by this mixture source. The contents of Ba and Mn in the topsoil were primarily contributed by industrial sources (Factor 3), accounting for 46.8% and 58.2% of their contents, respectively. Industrial sources also had large contributions to Co, Cu, Ni, Pb, Zn, and V, accounting for 27.8%, 32.2%, 31.7%, 20.5%, 38.1%, and 29.2% of their contents, respectively. For Mianyang topsoil, in general, natural sources, mixed sources of industry and traffic, and industrial sources contributed 32.6%, 34.4%, and 33.0% of the total HM contents, respectively.

### Spatial distribution of source contribution rates

**Figure 5:**
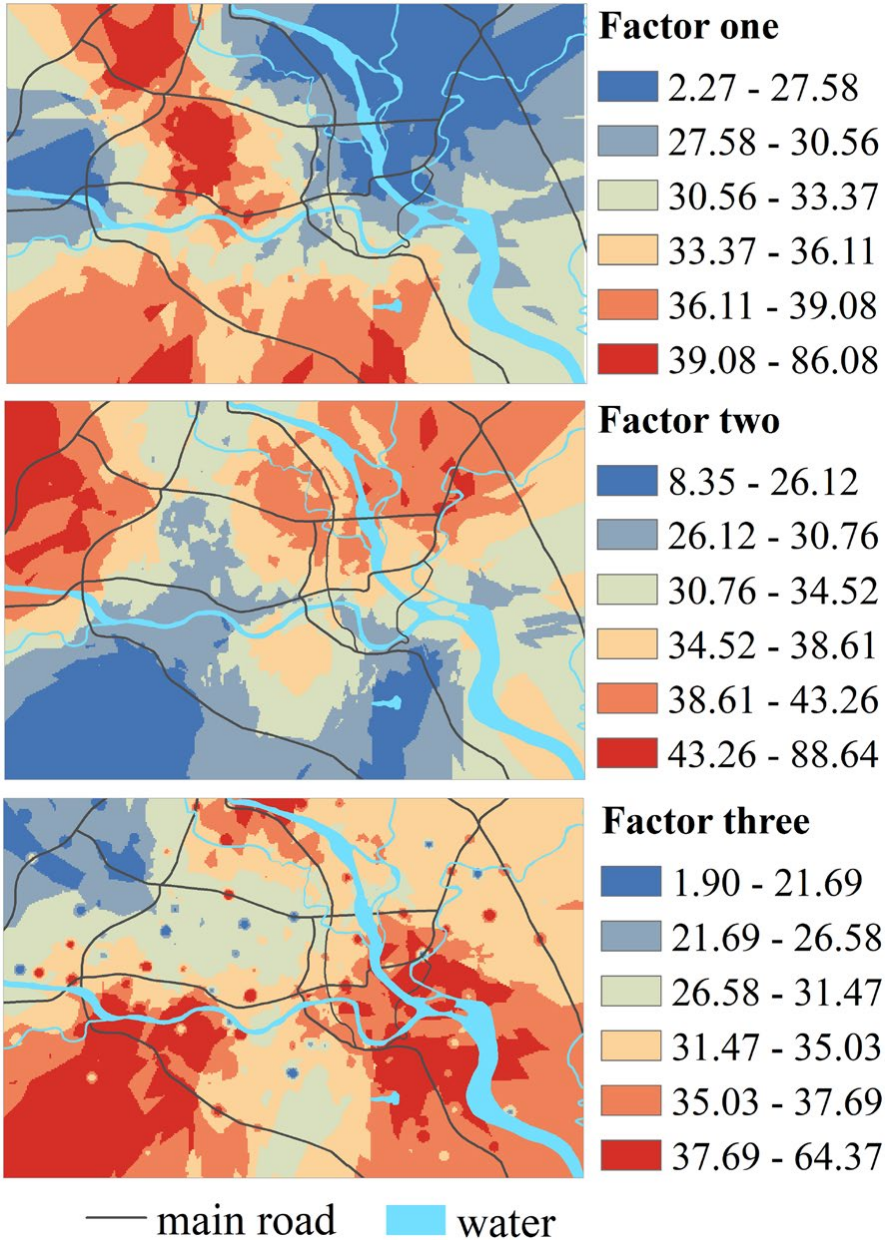
Spatial distribution of the contribution rate of the three sources [the figure was generated by Huaming Du using the ArcGIS 10.3 (https://developers.arcgis.com/)].

To further clarify the spatial distribution of different pollution sources and verify the sources of topsoil heavy metal pollution in Mianyang city, the spatial distribution of the contribution rates of the three sources was plotted by using OK, and the results are illustrated in Fig. 5.

The high contribution rate values of Factor 1 (natural source) were located at the southern edge and the center of the northern part of the study area. During the construction and development of this area, the soil was disturbed greatly, and the HMs in the parent rock were released, leading to a high concentration of HMs in the topsoil. The southern edge of the study area is near the urban fringe, and the disturbance of planting and disorderly construction activities to the soil led to an increase in HMs in the surrounding environment.

In general, the contribution rate of Factor 2 (mixed source of industry and traffic) in the northern part of the study area was higher than that in the southern part, and there were two high-value contribution rate areas of Factor 2. The first high-value area was distributed in the northwestern corner of the study area, which is a technology industrial area with electronics, machinery, and photoelectric technology. The second high-value area primarily appeared in the northeast of the study area with intensive traditional industrial activities. These two high-value areas all had trunk roads with heavy traffic. The distribution of the contribution rate from Factor 2 was approximately the opposite of that from Factor 1.

The contribution rate of Factor 3 (industrial source) varied considerably in space and showed a south-north distribution pattern. Overall, the contribution rate of Factor 3 in the south was greater than that in the north. The southwest part of the city is a high-tech industrial area with machinery plastics, energy, electronics, pharmaceuticals, and photoelectric technology; the southeast part of the city is an industrial area with machinery, petroleum, chemical industry, photoelectric technology, and power manufacturers.

## Conclusion

The levels of Ba, Cr, Cu, and Zn in the urban topsoil of Mianyang were significantly greater than their corresponding background values. The investigated HMs exhibited different spatial distribution characteristics due to the impacts of anthropogenic and natural factors. High Ba and Mn concentrations were found in the industrial zone. More industries and small reprocess shops were near the sites with high Cr contents. The high-value area of Cu was distributed in the high-tech industrial zone. Pb was mainly concentrated along urban trunk roads. The high-value sites of Zn were chiefly distributed in industrial zones. The HMs determined in the topsoil of Mianyang mainly originated from three main sources, viz. natural sources, mixed sources, and industrial sources, which accounted for 32.6%, 34.4%, and 33.0% of the total HM concentration, respectively. Industrial sources and mixtures of industry and traffic were the main anthropogenic sources of HMs in the urban topsoil of Mianyang, which should be considered by the local government. In view of the spatial distribution of these two anthropogenic sources, we suggest that the local government adopt differentiated measures in urban soil environmental protection and pollution source control: the northwestern and northeastern regions of the city should focus on controlling industrial discharges and traffic emissions, and the southwestern and southeastern regions of the city should focus on controlling industrial discharges. Due to the limitation of XRF, this work does not determine other toxic HMs, such as Cd and Hg, which will be investigated in future work. In addition, the speciation and bioavailability of HMs, as well as their ecological-health risks, will be further studied in the future.

## Data Availability

The datasets used and/or analyzed during the current study are available from the corresponding author on reasonable request.
